# The effectiveness of pharmaceutical interventions for obesity: weight loss with orlistat and sibutramine in a United Kingdom population-based cohort

**DOI:** 10.1111/bcp.12578

**Published:** 2015-05-22

**Authors:** Ian J Douglas, Krishnan Bhaskaran, Rachel L Batterham, Liam Smeeth

**Affiliations:** 1 Epidemiology Population Health, London School of Hygiene Tropical MedicineLondon, United Kingdom; 2 Centre for Obesity Research, Rayne Institute, Department of Medicine, University College LondonLondon, United Kingdom; 3 UCLH Centre for Weight Loss, Metabolic and Endocrine Surgery, University College London HospitalsLondon, United Kingdom

**Keywords:** electronic healthcare records, obesity, orlistat, pharmacoepidemiology, sibutramine

## Abstract

**Aims:**

Drug treatments for obesity have proven efficacy from randomized trials, but their effectiveness in routine clinical practice is unknown. We assessed the effects on weight and body mass index (BMI) of orlistat and sibutramine when delivered in routine primary care.

**Methods:**

We used United Kingdom data from the Clinical Practice Research Datalink to estimate the effects of orlistat or sibutramine on weight and BMI over 3 years following treatment initiation. For comparison, we matched each patient with up to five obese patients receiving neither drug. Mixed effects linear regression with splines was used to model change in weight and BMI. Mean change with 95% confidence intervals (CI) was estimated.

**Results:**

We identified 100 701 patients receiving orlistat, 15 355 receiving sibutramine and 508 140 non-intervention patients, with body mass index of 37.2, 36.6 and 33.2 kg m^−2^, respectively. Patients receiving orlistat lost, on average, 0.94 kg month^−1^ (0.93 to 0.95) over the first 4 months. Weight gain then occurred, although weight remained slightly below baseline at 3 years. Patients receiving sibutramine lost, 1.28 kg month^−1^ (1.26 to 1.30) over the first 4 months, but by 3 years had exceeded baseline weight. Non-intervention patients had slight increases in weight throughout the 3 year period, with gains ranging between 0.01 and 0.06 kg month^−1^.

**Conclusions:**

Orlistat and sibutramine had early effects on weight loss, not sustained over 3 years. As new treatments for obesity are approved, their effectiveness should be measured in routine clinical practice, as effectiveness may be considerably less than seen in randomized trials.

## What is Already Known about this Subject

- Drug treatments for obesity are licensed for use based on evidence of efficacy obtained from randomized clinical trials, but their effectiveness in routine clinical practice is generally unknown.
- In randomized clinical trials orlistat and sibutramine therapy led to reductions in weight varying between weight losses of between 4 and 10 kg over 12 months.

## What this Study Adds

- In routine clinical practice, orlistat and sibutramine, given for weight loss, are associated with a much reduced effect on weight when compared with randomized trials. No clinically meaningful impact was observed over 3 years following orlistat or sibutramine initiation in general practice in the United Kingdom.
- The effectiveness of approved drug treatments for obesity needs to be investigated in routine clinical practice as well as in randomized trials, as the effects seen in trials may not translate to real world use.

## Introduction

The prevalence of obesity and related health problems is increasing 1. Worldwide, over 500 million adults are obese with a body mass index (BMI) of 30 kg m^−2^ or more 2. Strategies to reduce weight range from transport policy and food labelling standards to lifestyle changes and targeted clinical interventions such as drug treatment and bariatric surgery. Currently, the only drug treatment with a license for use in obesity in Europe is orlistat. The license for sibutramine was suspended in 2010 for safety reasons. Although three new treatments (lorcaserin, phentermine/topiramate and bupropion/naltrexone) have been approved for use in the US, licenses were not obtained in Europe for lorcaserin and phentermine/topiramate with safety concerns highlighted 3,4 whilst European approval for bupropion/naltrexone is awaited.

United Kingdom (UK) and United States (US) guidelines generally recommend orlistat treatment for patients with a BMI of 30 kg m^−2^ or more (or 28 kg m^−2^ plus additional risk factors) and who have failed to achieve weight loss through other means 5,6. However, the evidence for effectiveness of drug interventions for obesity is based on the results of randomized trials and it is unknown how the efficacy measures in trials translate to effectiveness in general population-based healthcare. As a result, policy decisions may not be driven by the most relevant evidence. We therefore used data from the UK Clinical Practice Research Datalink (CPRD) to identify people receiving orlistat or sibutramine to determine their effects on weight and BMI over a 3 year period.

## Methods

### Clinical practice research datalink

The CPRD contains anonymized information from UK general practitioners including ∽8% of the UK population 7. Information includes complete recording of consultations, diagnoses, prescribed medicines and basic demographic data. Practices and patients are generally representative of the UK population 7 and data quality is subject to rigorous audits. The data have been used to conduct over 700 peer reviewed published studies and data validity is very good 8. Measures of BMI have recently been shown to be representative of national estimates in the UK 9.

### Study design

We conducted a longitudinal analysis of change in weight and BMI amongst incident orlistat and sibutramine users. Although the marketing authorization for sibutramine was suspended in 2010, we wanted to compare the results for orlistat with another pharmaceutical treatment for obesity. An obese, untreated patient group was also included.

### Study population, exposure and outcome

The study population was all patients registered with the CPRD before 31 Jan 2013 and with at least 12 months registration before a record of an orlistat or sibutramine prescription, ensuring they reflected incident interventions rather than continuing treatment. Use was determined by the recording of at least one orlistat or sibutramine prescription in prescribing files (see Appendix S1).

The date of first orlistat or sibutramine prescription was termed the index date and defined the start of follow-up. Follow-up ended at the earliest of 3 years later, death, a prescription for sibutramine prescription amongst orlistat recipients or orlistat amongst patients prescribed sibutramine, bariatric surgery, transfer from practice or last data recording date.

To provide an untreated group representative of patients meeting the UK guideline criteria for drug treatment for obesity 5, we matched each patient with up to five patients with a BMI of 30 kg m^−2^ or more, with at least 12 months follow-up by the index date of the intervention patient. Matches had to have no record of receiving orlistat, sibutramine or bariatric surgery prior to the index date, and were matched on age, gender and general practice. For each patient the closest recorded weight to the index date was identified. Patients in the comparison group tended not to have a recent weight measure on their matched index date, and we anticipated weight may change substantially over a long period. To avoid errors associated with using out-dated measures 10, follow-up for this group was started at the weight measured nearest to index and ended at the earliest of 3 years later, death, bariatric surgery, first orlistat or sibutramine prescription, transfer from practice or last data recording date. The purpose of this group was to assess the stability of recorded weight measures in the CPRD for an obese population not receiving drug treatment or surgery for obesity. *Post hoc* we found that patients in this comparison group tended to have a lower baseline BMI than patients in the drug treated groups; We therefore further stratified this group into high and low baseline BMI, based on whether the index BMI was above or below the median for the no-intervention group.

Height and weight records were extracted from clinical files. Implausible records for obese adults were discarded (any weight <40 kg or >300 kg; <1% all recorded weight measures). Weights between 225 and 300 kg were discarded if other measures on the same day were <225 kg or where the ratio to other recorded weights for the individual was >1.5 (<0.01% of all recorded weight measures). For patients with recorded height, BMI was calculated for each weight record. For orlistat and sibutramine recipients the nearest weight prior to index date was taken as baseline weight. Records indicating the general practitioner had given advice about diet or physical activity, or that the patient had been referred to other specialist settings to help manage obesity were also extracted from patient's clinical and referral files.

### Statistical analysis

Change in weight and BMI was modelled using mixed effects linear regression. As weight was anticipated to be non-linear over follow-up, splines were fitted to account for different phases of weight change. A linear spline model was fitted facilitating simple interpretation of weight/BMI changes over time; the Akaike Information Criterion (AIC) was used to determine the optimal number and time point of spline knots. The first knot after index was determined by fitting every possible knot in steps of 1 month and selecting the model with the lowest AIC. Further knots were added in the same way until AIC increased or decreased by a negligible amount (<4 units). From the final model, weight/BMI and 95% confidence intervals (CI) for the study population were estimated over 3 years. Separate analyses were conducted in patients with diabetes or cardiovascular disease because of the specific importance of weight reduction therapies among these groups 6.

### Sensitivity analyses and other considerations

Anticipating many patients would not continue orlistat or sibutramine treatment for the full 3 year period, we conducted a sensitivity analysis censoring follow-up at evidence of a treatment break. Prescriptions were estimated to last 30 days and if no further prescription was recorded within the subsequent 90 days, a treatment break was inferred, triggering censoring at the end of 90 days. We also conducted analyses censoring at 01 May 2009 when orlistat became available without prescription, and excluding patients receiving a single orlistat or sibutramine prescription, anticipating this group included patients who never took either drug.

To determine whether changes in weight over time were biased by preferentially observing later measures for patients whose baseline weight systematically differed from the group as a whole, the baseline weight of patients contributing measures beyond 1 and 2 years was compared with the baseline weight of all patients.

### Ethics

Approval was obtained from the Medicines and Healthcare products Regulatory Agency's Independent Scientific Advisory Committee and ethical approval granted by the London School of Hygiene and Tropical Medicine ethics committee.

## Results

Table1 shows background details of all patients included. One hundred thousand seven hundred and one patients received orlistat, 15 355 received sibutramine and 508 140 had no intervention. Mean age was 46, 44 and 46 years for orlistat, sibutramine and no-intervention patients, respectively, and the majority were women (>75%). Follow-up post-baseline varied, with orlistat recipients followed on average for 4.9 years compared with 5.7 years for patients prescribed sibutramine and 4.5 years amongst no-intervention patients. BMI at baseline varied slightly, with patients receiving orlistat having a mean BMI of 37.2, compared with 36.6 in sibutramine recipients and 33.2 in the no-intervention group. Cerebrovascular disease, coronary heart disease, hypertension, type 2 diabetes and statin use were more common amongst orlistat than sibutramine recipients, possibly reflecting the known cardiovascular safety concerns with sibutramine.

**Table 1:** Background details of study population

	Orlistat (*n* = 100 701)	Sibutramine (*n* = 15 355)	No clinical intervention (*n* = 508 140)
Age, mean years (SD)	46.2 (14.0)	43.5 (13.1)	46.4 (14.1)
Female, *n* (%)	76 946 (76.4)	12 560 (81.8)	380 389 (74.9)
Follow-up post-index, mean years (SD)	4.9 (3.2)	5.7 (2.9)	4.5 (3.2)
Last BMI before index missing			
Mean (SD)	37.2 (6.4)	36.6 (6.6)	33.2 (5.1)
Number (%)	2456 (2.4)	489 (3.1)	6904 (1.3)
Time to index, days (SD)	105 (472)	144 (572)	0
Comorbidities, *n* (%)			
CVD	2344 (2.3)	177 (1.2)	9455 (1.9)
CHD	7243 (7.2)	475 (3.1)	25 141 (5.0)
Hypertension	29 501 (29.3)	2786 (13.3)	115 232 (22.7)
T2D	17 938 (17.8)	2072 (13.5)	48 569 (9.6)
Statin use	22 002 (21.9)	2041 (13.3)	73 094 (14.4)
Weight loss advice given prior to start of study follow-up	46 147 (45.8)	6270 (40.8)	125 317 (24.7)

CHD, coronary heart disease; CVD, cerebrovascular disease; T2D, type 2 diabetes.

**Figure 1:**
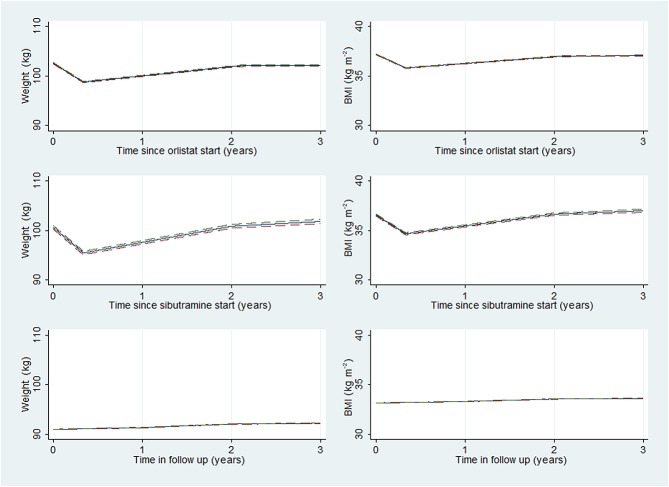
Estimated mean change in weight and BMI (with 95% confidence intervals) over 3 years following initiation of orlistat, sibutramine or no intervention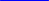, mean; 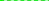, upper 95% confidence interval; 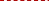, lower 95% confidence interval.

To be included in the analysis, patients had to have at least one pre-index recorded weight. This was available for 99 420 orlistat recipients (99%), 15 060 sibutramine recipients (98%) and 505 790 patients with no intervention (99%). Estimated changes in weight and BMI over the 3 year follow-up are shown in Table 2 and Figure 1.

### Orlistat

Amongst patients receiving orlistat, weight loss in the first 4 months was 0.94 kg month^−1^ (0.93–0.95), followed by an increase between 5–25 months of 0.16 kg month^−1^ (95% CI 0.15, 0.16) and an increase of 0.01 kg month^−1^ (95% CI 0.00, 0.02) between 26 and 36 months (Table2, Figure1). With censoring at orlistat treatment breaks, results were similar (Table2). Weight changes in patients receiving orlistat with cardiovascular disease were similar to those seen in the orlistat group as a whole, but patients with diabetes appeared to have a reduced level of initial weight loss, with −0.78 kg year^−1^ during the initial 4 months (Table 2).

**Table 2:** Rate of change in weight and BMI over 3 year follow-up

Time in follow-up	*n*	Estimated weight change, kg month^−1^ (95% CI)	Estimated BMI change, kg m^−2^ month^−1^ (95% CI)
Orlistat			
1–4 months	99 420	−0.94 (−0.93, −0.95)	−0.34 (−0.34, −0.34)
5–25 months	74 000	0.16 (0.15, 0.16)	0.06 (0.06, 0.06)
26–36 months	37 487	0.01 (0.00, 0.02)	0.01 (0.00, 0.01)
Orlistat censoring at end of therapy			
1–4 months	99 420	−1.01 (−1.00, −1.02)	−0.37 (−0.37, −0.37)
5–25 months	57 039	0.10 (0.10, 0.11)	0.04 (0.04, 0.04)
26–36 months	20 813	−0.03 (−0.02, −0.04)	−0.01 (−0.01, −0.01)
Orlistat and cardiovascular disease			
1–4 months	32 747	−0.97 (−0.95, −0.98)	−0.35 (−0.34, −0.35)
5–25 months	27 428	0.13 (0.12, 0.13)	0.05 (0.04, 0.05)
26–36 months	16 358	0.00 (−0.01, 0.01)	0.00 (0.00, 0.00)
Orlistat and T2D			
1–4 months	17 836	−0.78 (−0.77, −0.80)	−0.28 (−0.27, −0.29)
5–25 months	15 687	0.10 (0.09, 0.11)	0.04 (0.03, 0.04)
26–36 months	10 351	−0.03 (−0.02, −0.04)	−0.01 (−0.01, −0.02)
Sibutramine			
1–4 months	15 060	−1.28 (−1.26, −1.30)	−0.47 (−0.46, −0.48)
5–24 months	11 130	0.27 (0.26, 0.28)	0.10 (0.10, 0.10)
25–36 months	5406	0.08 (0.06, 0.10)	0.03 (0.02, 0.04)
Sibutramine censoring at end of therapy			
1–4 months	15 060	−1.30 (−1.28, −1.32)	−0.48 (−0.47, −0.49)
5–24 months	9382	0.23 (0.22, 0.25)	0.08 (0.08, 0.09)
25–36 months	3590	0.00 (−0.02, 0.03)	0.00 (−0.01, 0.01)
Sibutramine and cardiovascular disease			
1–4 months	3043	−1.12 (−1.07, −1.17)	−0.41 (−0.40, −0.43)
5–24 months	2454	0.19 (0.17, 0.21)	0.07 (0.06, 0.08)
25–36 months	1411	0.13 (0.10,.0.17)	0.05 (0.03, 0.06)
Sibutramine and T2D			
1–4 months	2047	−0.94 (−0.89, −0.99)	−0.34 (−0.32, −0.36)
5–24 months	1764	0.18 (0.16, 0.20)	0.07 (0.06, 0.07)
25–36 months	1130	0.02 (−0.01, 0.06)	0.01 (0.00, 0.02)
No intervention			
1–12 months	505 790	0.03 (0.03, 0.03)	0.01 (0.01, 0.01)
13–24 months	193 235	0.06 (0.06, 0.06)	0.01 (0.01, 0.02)
25–36 months	125 823	0.02 (0.02, 0.02)	0.00 (0.00, 0.01)

Mean baseline weight for patients receiving orlistat was 103 kg (SD = 21 kg) and mean baseline weight and SD of patients contributing weight measures after 4 months (*n* = 79 297) and 25 months (*n* = 54 150) was identical.

Sensitivity analyses excluding patients given single orlistat prescriptions (*n* = 27 932) and censoring at 01 May 2009 had no material effect on the results (Supporting Information).

### Sibutramine

Amongst patients receiving sibutramine, weight loss in the first 4 months was 1.28 kg month^−1^ (95% CI 1.26, 1.30), followed by an increase between 5–24 months of 0.27 kg month^−1^ (95% CI 0.26, 0.28) and an increase of 0.08 kg month^−1^ (95% CI 0.06, 0.10) between 25 and 36 months (Table 2, Figure1). With censoring at sibutramine treatment breaks, results were similar (Table2). Weight changes in patients receiving sibutramine with cardiovascular disease were slightly reduced in the first four months compared with the sibutramine group as a whole (1.12 kg month^−1^), and patients with diabetes appeared to have a further reduced level of initial weight loss, with −0.94 kg month^−1^ during the initial 4 months (Table2).

Mean baseline weight for patients prescribed sibutramine was 101 kg (SD = 21 kg) and mean baseline weight and SD of patients contributing weight measures after 4 months (*n* = 11 130) and 24 months (*n* = 5406) was identical.

Sensitivity analyses excluding patients given single sibutramine prescriptions (*n* = 3577) and censoring at 01 May 2009 had no material effect on the results (see Table S1).

### No intervention

People without intervention had relatively small, gradual increases in weight and BMI throughout the 3 year period (Table2, Figure1). In the *post hoc* analysis stratified by baseline BMI, patients with a lower baseline BMI gained weight throughout follow-up whilst those with higher baseline BMI had negligible changes in weight during the 3 year period (see Table S2).

### BMI

As expected, the pattern for BMI closely followed that for weight in all analyses (Table2, Figure 1).

## Discussion

We assessed the effects on weight and BMI of drug interventions for obesity delivered in routine clinical practice among the general population. Orlistat treatment in patients with an average BMI of 37.2 kg m^−2^ was followed by a small reduction in weight and BMI over 4 months, with gradual reversal of these changes over 3 years. Our findings suggest an average patient trajectory would involve a reduction in weight of 2.5 kg 1 year after orlistat initiation, with gains of 1.9 kg in year 2 and 0.3 kg in year 3, resulting in an overall reduction of 0.3 kg by the end of year 3. The corresponding average 3 year reduction in BMI was 0.14 kg m^−2^. Sibutramine therapy was followed by an initial weight loss, on average 3.0 kg in year 1, but an increase of 3.2 kg in year 2 and a further 0.9 kg in year 3 was seen, resulting in an overall gain of 1.2 kg or an increase in BMI of 0.48 kg m^−2^. Untreated patients had slight increases in weight over the 3 year period, gaining on average 1.2 kg. Although orlistat appears to have been slightly more effective than sibutramine, neither drug achieved clinically relevant levels of long term weight loss.

In this population based sample, 97% of orlistat and 94% of sibutramine recipients had a BMI of 28 kg m^−2^ or more, with 91% and 88%, respectively, over 30 kg m^−2^, suggesting treatment largely concords with national guidelines 5,6. The non-intervention group was selected as being representative of patients eligible to receive drug treatment for obesity, but tended to have a lower baseline BMI than those treated with sibutramine or orlistat, The very small changes in weight/BMI observed in this group highlight the stability and utility of weight measures recorded in the CPRD, and therefore their suitability for investigating the impact of interventions for obesity.

### Strengths and weaknesses

To our knowledge, this is the largest study examining the effectiveness of drug treatment for obesity delivered in a population-based healthcare system. The data we used are representative of the UK population and reflect current medical practice, and so the results are likely to generalize to the UK population as a whole.

We recently demonstrated that CPRD recorded BMI has good concordance with Health Survey for England at the general population level when restricted to patients with recently measured BMI (within 3 years) 9. For our study, measures were applied on the date taken, except baseline weight for the orlistat and sibutramine groups, where the most recent pre-intervention measure was used. In both cases the average time from measurement to intervention was less than 1 year and we therefore do not anticipate major bias in our measures of weight.

There was variability in the number of weight recordings per patient. The mean number of measures over 3 years was six for both orlistat and sibutramine recipients, suggesting good longitudinal measures are available in this population. One concern was that people with weight measures at later time points may not represent the intervention group as a whole, and could bias estimates of weight change over time. We found no difference in baseline weight between those with recorded weight in years 2 and 3, and the group as a whole, but cannot exclude the possibility that weight was preferentially recorded for patients who responded to the intervention in a way that was not representative.

Current guidelines in the UK recommend drug treatments for obesity should only be given in combination with advice about diet and physical activity 5. We were able to confirm that such advice or relevant secondary referrals had been made for ∽50% of our study population, but were unable to confirm whether patients attended secondary referrals, or whether similar advice had been given to other patients but not recorded. In the absence of such information we cannot estimate separately the effects of lifestyle advice from those of drug treatment.

### Comparison with previous studies

#### Orlistat

The effects of orlistat on weight in randomized trials have been modest, with an additional loss of 4.12 kg at 12 months compared with placebo 10. Absolute weight loss in trials has been 7–10 kg over 1 year with some weight regain in the subsequent year 11–13. Previous evidence from non-randomized settings is limited with a single study based on Health Maintenance Organization data from Israel 14. Orlistat adherence was poor with 2% completing 12 months therapy and estimates of weight loss for orlistat recipients were unavailable.

#### Sibutramine

In randomized trials, sibutramine has been associated with absolute weight loss varying from 4 to 8 kg over a 12 month period, with a lack of long term evidence from routine clinical use 15–17. One observational study detected a loss of up to 10 kg, but was restricted to a 12 week follow-up and it is unknown whether this effect was sustained for a longer period 18.

Our findings suggest that results from carefully monitored trials may not generalize well to real world treatment, with both orlistat and sibutramine appearing to be much less effective in general practice. Some of this difference may be a result of shorter treatment courses in general usage; 28% of orlistat and 23% of sibutramine patients only received a single prescription, indicating they received little or no treatment. Furthermore, only 22% received orlistat in year 2 and 13% in year 3. For sibutramine, 18% received prescriptions in year 2 and 10% in year 3. The median proportion of time in follow-up over which orlistat and sibutramine were taken was 11% and 14%, respectively, based on prescribing records. This compares with ∽50% treatment adherence at 2 years in trials. Treatment duration in general practice is likely to reflect individual patient and physician assessment of the risk/benefit balance, and most patients do not receive orlistat for long periods. Nonetheless when censoring follow-up at orlistat withdrawal, changes in weight remained lower than seen in trials, with overall reductions of 2.2 kg and 1.3 kg for orlistat and sibutramine, respectively, in 3 years, suggesting shorter treatment duration does not wholly explain the differences between performance in trials *vs*. general practice. Whilst some individuals may gain greater benefits from medication use, these results do not suggest any long term clinically relevant benefit for current and recently available obesity medication when given at the population level.

In conclusion, new drug treatments for obesity are approved based on a positive evaluation of their benefits and risks as demonstrated in randomized clinical trials. However, patients, prescribers and policy makers need to know how effective a treatment might be in the routine clinical setting in order to inform therapy decisions. We have shown that pharmaceutical anti-obesity interventions delivered through a population-based healthcare system may lead to a substantially lower degree of weight loss than was seen in randomized trials. This highlights the need for a post-approval evaluation of the effectiveness of anti-obesity treatments as delivered in routine clinical care.

## Competing Interests

All authors have completed the Unified Competing Interest form at http://www.icmje.org/coi_disclosure.pdf (available on request from the corresponding author) and declare IJD had support from the Medical Research Council, LS had support from the Wellcome Trust for the submitted work, IJD had personal fees and shareholdings in GlaxoSmithKline and personal fees from Gilead for work unrelated to weight loss, LS had personal fees from GlaxoSmithKline for work unrelated to weight loss in the previous 3 years and there are no other relationships or activities that could appear to have influenced the submitted work.

IJD is funded by a Medical Research Council Methodology Fellowship, KB is funded by a National Institute for Health Research postdoctoral fellowship and LS is funded by a Wellcome Trust Fellowship. RB is funded by the Rosetrees’ Trust. The funders played no role in the design and conduct of the study, collection, management, analysis and interpretation of the data and preparation, review or approval of the manuscript.

## Contributors

Ian Douglas conceived and designed the study, obtained and analyzed the data, interpreted results and drafted the manuscript. Krishnan Bhaskaran conceived and designed the study, obtained and analyzed the data, interpreted results and drafted the manuscript. Rachel Batterham conceived and designed the study, interpreted results and drafted the manuscript. Liam Smeeth conceived and designed the study, interpreted results and drafted the manuscript. Ian Douglas takes full responsibility for the work as a whole, including the study design, access to data, and the decision to submit and publish the manuscript.

## Guarantor statement

Ian Douglas is the guarantor of this work and, as such, had full access to all the data in the study and takes responsibility for the integrity of the data and the accuracy of the data analysis.

## Supporting Information

Supporting info item

Supporting info item
