# Supplementary figures and images for: The effectiveness of pharmaceutical interventions for obesity: weight loss with orlistat and sibutramine in a United Kingdom population-based cohort
Source: Br J Clin Pharmacol. 2015 May 22;79(6):1020–7. doi: 10.1111/bcp.12578 (PMC4456134; doi:10.1111/bcp.12578)

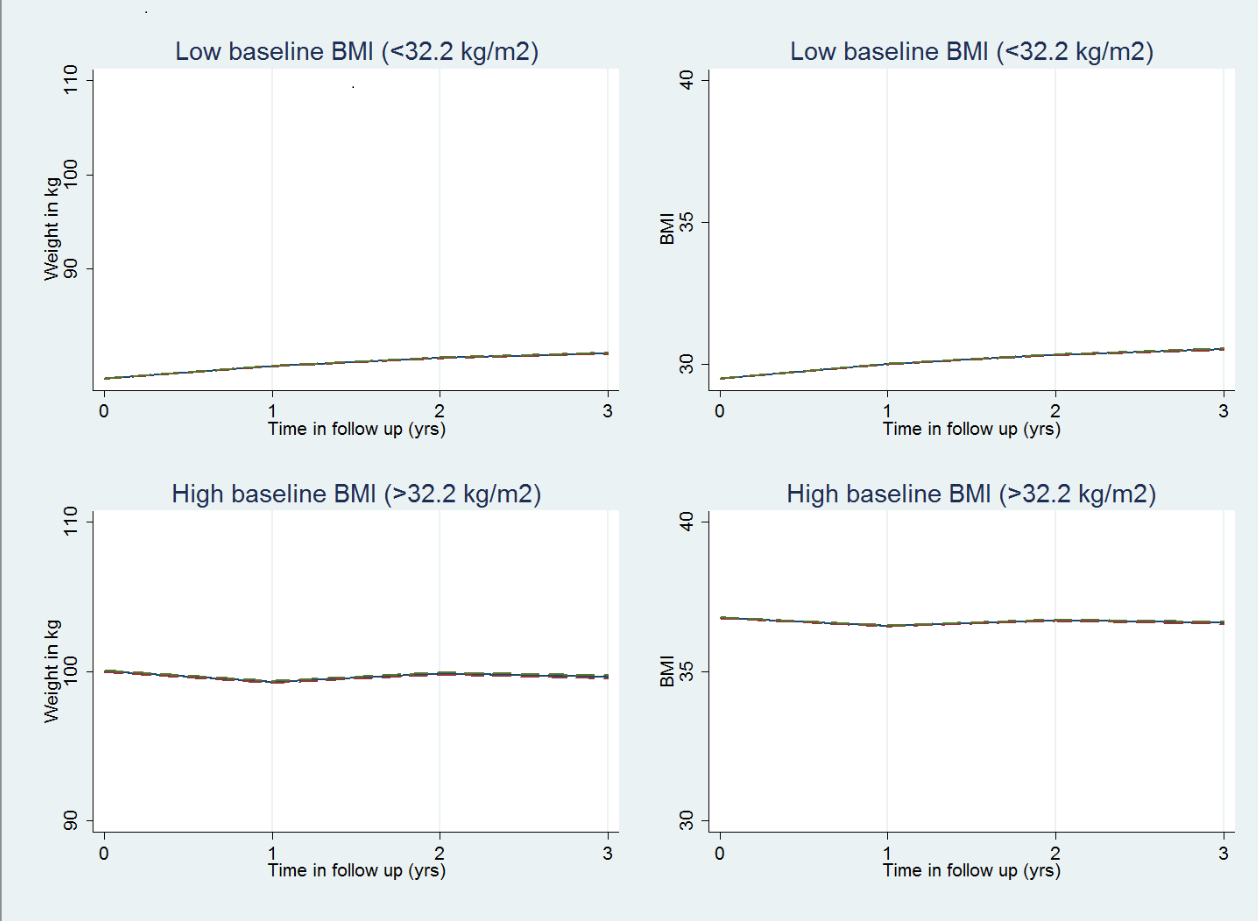

Supplement: Supplementary file 1 [file bcp0079-1020-sd1.png]
