# Supplementary material for: The effectiveness of pharmaceutical interventions for obesity: weight loss with orlistat and sibutramine in a United Kingdom population-based cohort
Source: Br J Clin Pharmacol. 2015 May 22;79(6):1020–7. doi: 10.1111/bcp.12578 (PMC4456134; doi:10.1111/bcp.12578)
Supplement: Supplementary file 2 [file bcp0079-1020-sd2.docx]

**Web Appendix 1: Code lists for data extraction**

Code list 1: Orlistat

*prodcode* *drugsubstance*

525 orlistat

3020 orlistat

39843 orlistat

40337 orlistat

Code list 2: Sibutramine

*prodcode drugsubstance*

699 sibutramine hydrochloride

803 sibutramine hydrochloride

5504 sibutramine hydrochloride

5625 sibutramine hydrochloride

**eTable 1: Supplementary analyses**

| Time in Follow Up | Estimated weight change, Kg/month (95% CI) |
| --- | --- |
| Orlistat censored at 01 May 2009 (n=69,583) |  |
| 1-4 months | -0.95 (-0.94 to -0.96) |
| 5-12 months | 0.18 (0.17 to 0.18) |
| 13-36 months | 0.03 (0.02 to 0.04) |
| Orlistat excluding single prescription patients (n=71,488) |  |
| 1-4 months | -1.06 (-1.05 to -1.07) |
| 5-12 months | 0.17 (0.16 to 0.17) |
| 13-36 months | 0.03 (0.02 to 0.04) |
| Sibutramine censored at 01 May 2009 (n=13,636) |  |
| 1-4 months | -1.28 (-1.26 to -1.31) |
| 5-12 months | 0.27 (0.26 to 0.29) |
| 13-36 months | 0.09 (0.07 to 0.12) |
| Sibutramine excluding single prescription patients (n=11,483) |  |
| 1-4 months | -1.42 (-1.39 to -1.44) |
| 5-12 months | 0.28 (0.27 to 0.29) |
| 13-36 months | 0.11 (0.09 to 0.13) |

**eTable 2: Stratified analysis of non-intervention group**

| Time in Follow Up | N | Estimated weight change, kg/month (95% CI) | Estimated BMI change, kg/m^2^/month (95% CI) |
| --- | --- | --- | --- |
| **Low Baseline BMI (<32.2 kg/m^2^)** | | | |
| 1-12 months | 249,248 | 0.12 (0.12 to 0.12) | 0.04 (0.04 to 0.04) |
| 13-24 months | 100,702 | 0.07 (0.07 to 0.08) | 0.03 (0.03 to 0.03) |
| 25-36 months | 67,043 | 0.04 (0.04 to 0.05) | 0.02 (0.01 to 0.02) |
| **High Baseline BMI (>32.2 kg/m^2^)** | | | |
| 1-12 months | 256,542 | -0.06 (-0.06 to -0.06) | -0.02 (-0.02 to -0.02) |
| 13-24 months | 92,535 | 0.05 (0.04 to 0.05) | 0.02 (0.01 to 0.02) |
| 25-36 months | 58,789 | -0.02 (-0.01 to -0.02) | -0.007 (-0.005 to -0.01) |
